# Small Intestinal Adenocarcinoma in a Patient With Celiac Disease

**DOI:** 10.7759/cureus.13039

**Published:** 2021-01-31

**Authors:** Mohammad Abudalou, Ali F Al Sbihi, Aleksandr Perepletchikov, Christopher Stallwood

**Affiliations:** 1 Internal Medicine, St. Elizabeth's Medical Center, Brighton, USA; 2 Internal Medicine, Detroit Medical Center (DMC) Sinai-Grace Hospital, Detroit, USA; 3 Pathology, St. Elizabeth's Medical Center, Brighton, USA; 4 Gastroenterology, St. Elizabeth's Medical Center, Brighton, USA

**Keywords:** celiac disease, small intestinal tumours

## Abstract

Celiac disease (CD) is a systemic immune-mediated disorder against gluten, leading to an autoantibody response causing damage to the small intestinal mucosa. CD has been associated with gastrointestinal malignancies, most commonly gastrointestinal lymphoma. Rare malignancies have also been reported, such as small intestinal adenocarcinoma. In this report, we present a case of a 91-year-old male with a history of CD, noncompliant with a gluten-free diet, who presented with weight loss, abdominal pain, and gastrointestinal bleeding secondary to a newly discovered adenocarcinoma of the jejunum.

## Introduction

Celiac disease (CD) occurs in genetically susceptible individuals due to an environmental trigger called gluten, which is derived from wheat. Gluten contains gliadin, which is resistant to degradation in the intestine. When gliadin traverses the small intestinal epithelial barrier in a patient with CD, a pro-inflammatory immune reaction is triggered. This leads to small intestinal mucosal injury, and ultimately, malabsorption. Weight loss and chronic diarrhea are often the presenting symptoms. The mean prevalence is one to two percent in the general population [1-2]. Small intestinal adenocarcinoma is a rare malignancy occurring in 0.6-0.7 per 100,000 of the general population per year [3]. There is an increased risk of this tumor in patients with CD [4]. We report a case of adenocarcinoma of the jejunum in a male patient with CD, followed by a detailed discussion of CD and its association with this tumor.

## Case presentation

**Figure 1:**
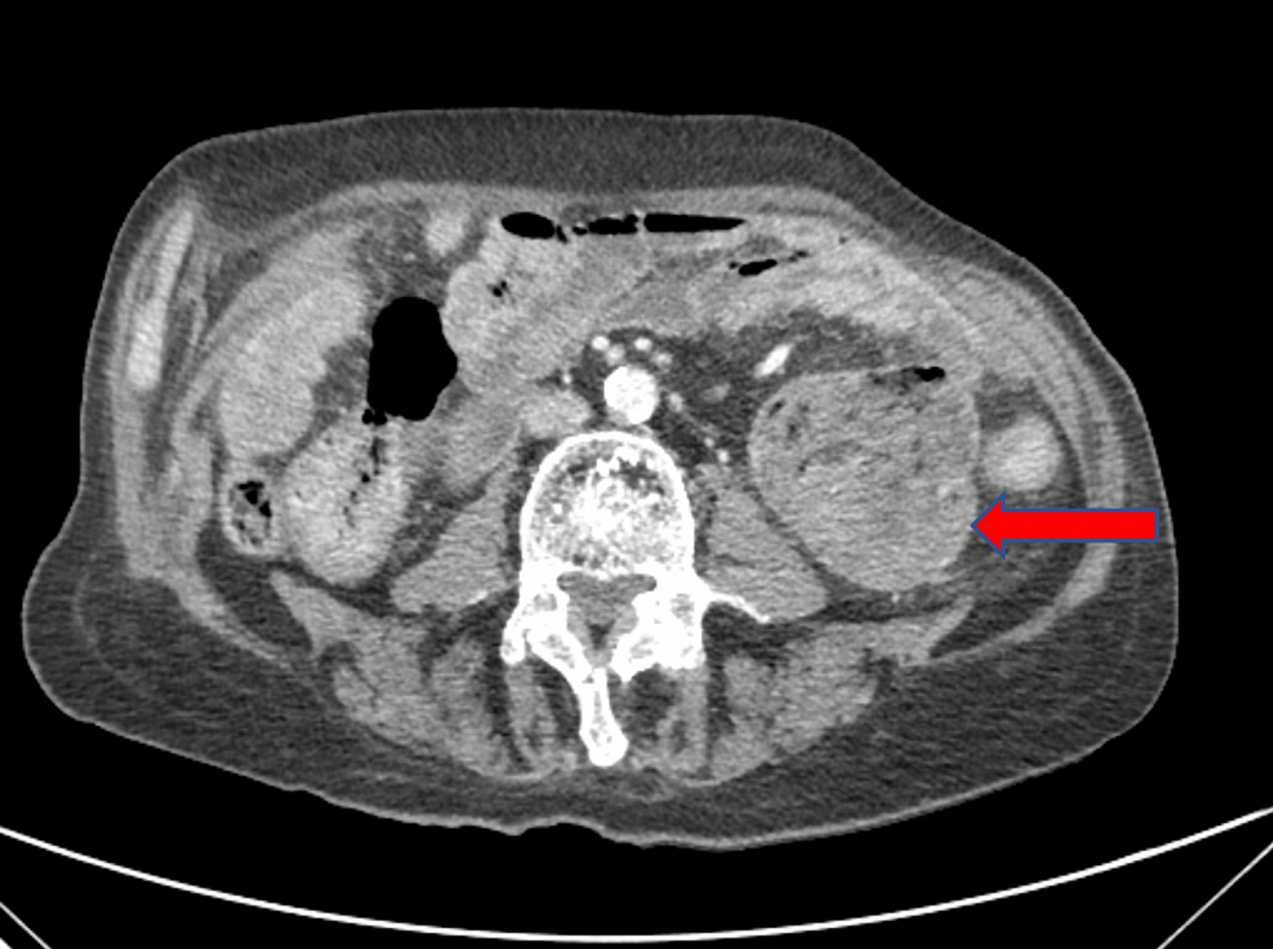
CT of the abdomen and pelvis. Red arrow: proximal jejunum is dilated up to 6.8 cm in diameter.

A 91-year-old male with hypertension, coronary artery disease, and a longstanding history of CD, noncompliant with a gluten-free diet, presented to the ED for evaluation of intermittent upper abdominal pain and unintentional weight loss of 10 pounds over a period of two months. His vital signs were initially normal, however, after an episode of hematemesis followed by hematochezia, he became hypotensive to 80/60 mmHg and tachycardic to 100 beats/min. Physical examination was notable for pale conjunctiva and tenderness to palpation in the upper abdomen. Laboratory evaluation revealed a white blood cell count of 13.2 x 103/uL (reference (R): 4.5-11.0 x 103/uL), hemoglobin of 5.9 g/dL (R: 12-17 g/dL), blood urea nitrogen of 34 mg/dL (R: 5-25 mg/dL), and creatinine of 0.8 mg/dL (R: 0.6-1.8 mg/dL). CT of the abdomen and pelvis demonstrated a 6.8 cm dilated proximal jejunum (Figure 1). 

**Figure 2:**
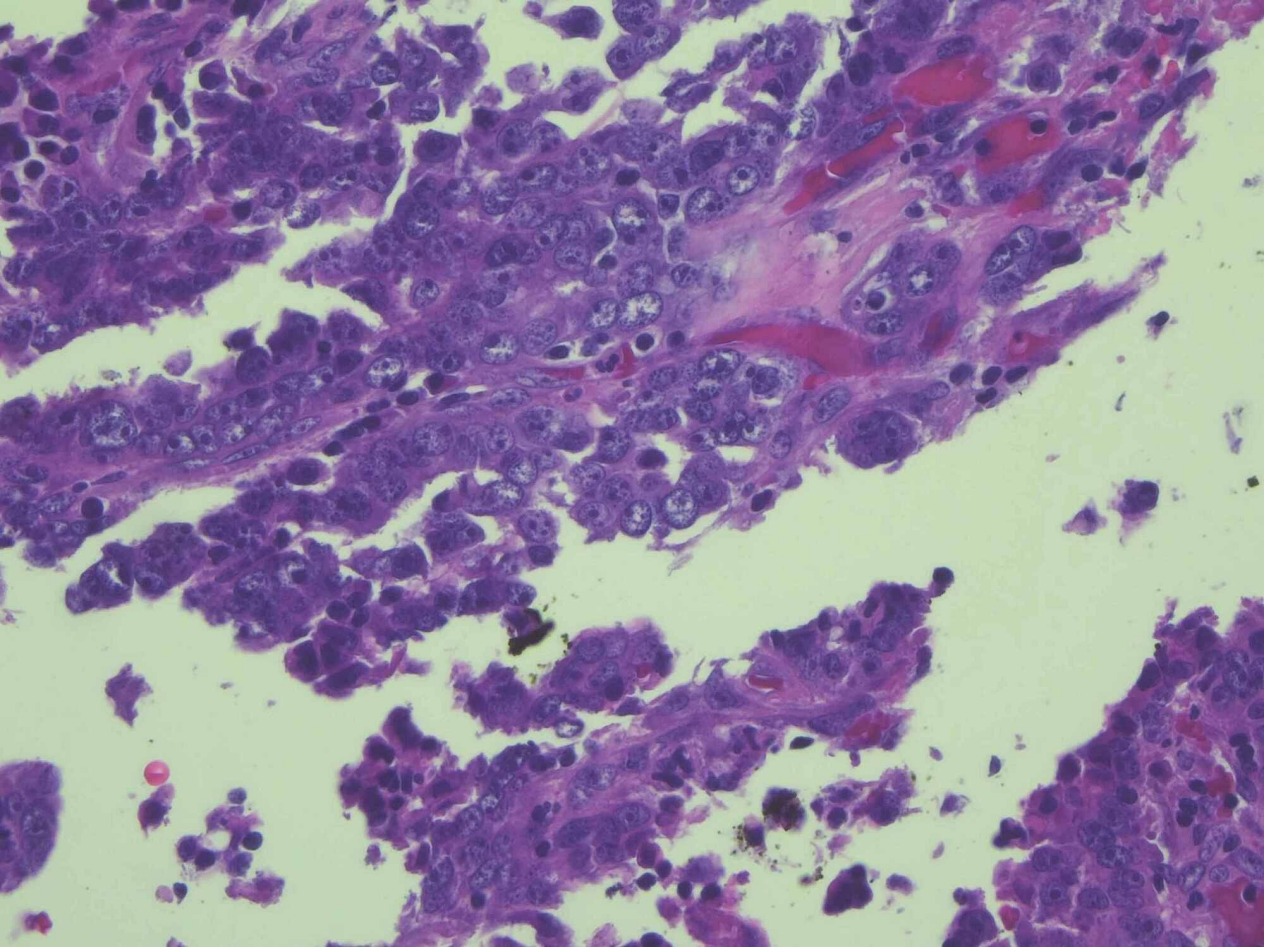
Carcinoma with glandular formation, 40x magnification.

**Figure 3:**
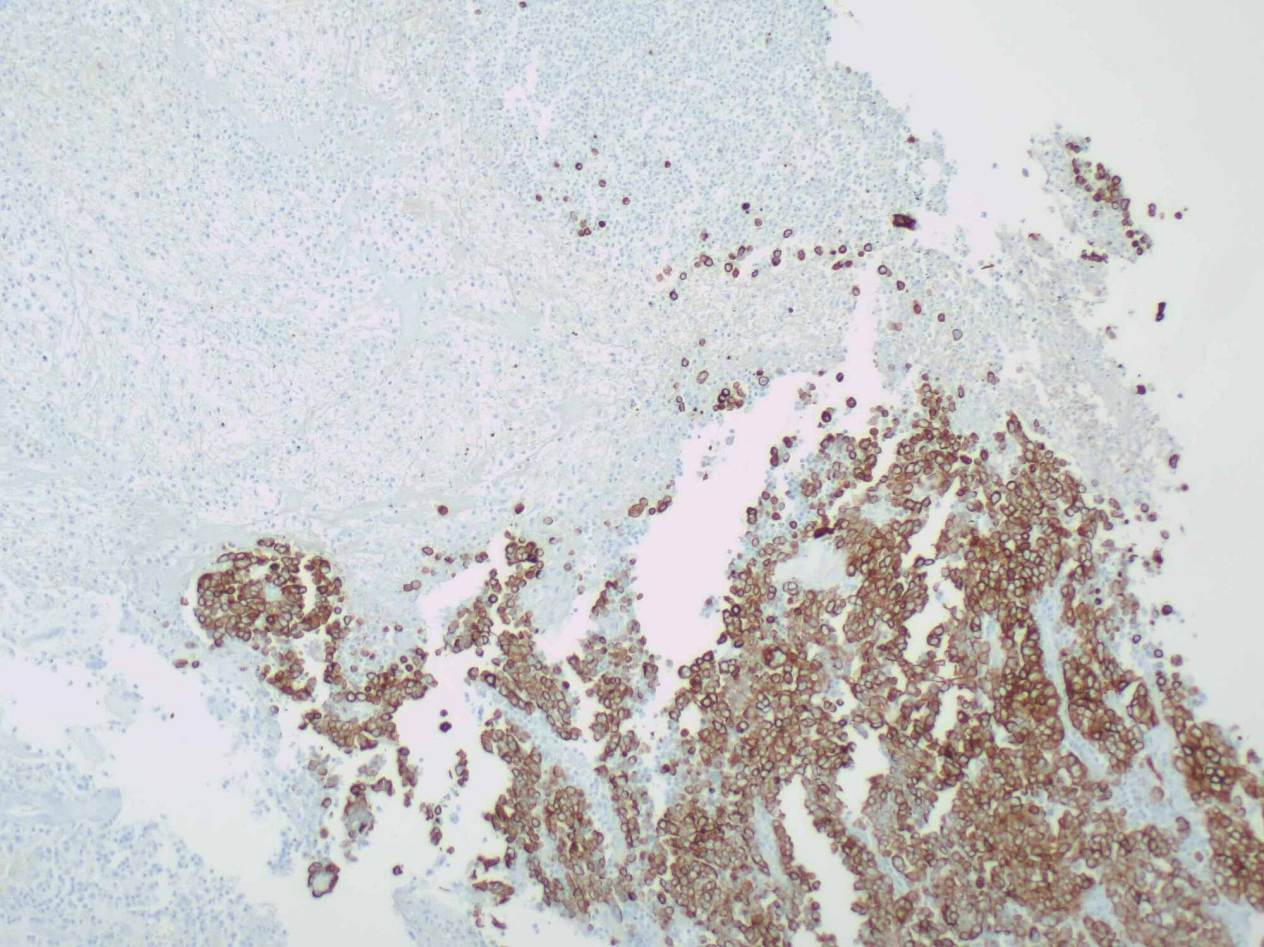
Cytokeratin immunoreactivity.

The patient was resuscitated with four units of packed red blood cells and two liters of normal saline. Intravenous pantoprazole was administered. Surgery was consulted and a nasogastric tube was placed for gastric decompression. Small bowel enteroscopy discovered two nonbleeding superficial clean-based ulcers in the duodenum and an ulcerated jejunal mass, which appeared to be the source of bleeding. Biopsies from the mass showed an invasive, poorly differentiated adenocarcinoma. Subsequently, exploratory laparotomy revealed additional masses in the ileum and transverse colon suspicious for malignancy; thus, partial small bowel and partial colonic resection with end colostomy and Hartmann’s pouch were performed. The mass in the jejunum measured 1.7 cm x 1 cm x 0.2 cm. Pathologic evaluation revealed invasive, poorly differentiated carcinoma with gland formation only in the lesion resected from jejunum (Figure 2). Cytokeratin was immunoreactive (Figure 3). CK7, CK20, TTF-1, LCA, CD56, synaptophysin, and chromogranin stains were negative. Regional resected lymph nodes and other resected lesions were devoid from cancer. The American Joint Committee of Cancer (AJCC) stage was pT2N0. The patient’s post-operative course was uncomplicated, and he was discharged with outpatient oncology follow up. On follow up visit four weeks after surgery, the patient was recovering well and without any complaints. He was offered chemotherapy, but declined any treatment. 

## Discussion

Celiac disease is an immune-mediated reaction to gliadin, a component of gluten [2]. There is a known genetic predisposition associated with CD. The predisposing genes are primarily HLA-DQ2 (DQA1*05-DQB1*02) and HLA-DQ8 (DQA1*03-DQB1*0302). Various other non-HLA genes are thought to contribute to the development of CD in some populations [2]. CD has been associated with multiple gastrointestinal malignancies, including colon, esophageal and small bowel cancer. Small intestinal adenocarcinoma arises most commonly in the duodenum (54.8%), but also in the jejunum (29.9%) and the ileum (15.3%) [5]. It occurs 50 times less frequently than colorectal adenocarcinoma and it follows a distinct molecular pathway for tumorigenesis [6-7]. Environmental factors, such as alcohol intake and diet high in red meat, sugar and starch are associated with an increased risk of this tumor [8]. CD increases the risk of small intestinal adenocarcinoma, which most commonly develops in misdiagnosed CD patients or in CD patients who are noncompliant with a gluten-free diet [9]. In a recent cohort study of 48,119 individuals with CD, 29 patients (0.02%) developed small intestinal adenocarcinoma one year after diagnosis and the hazard ratio of developing the tumor was highest during the first 10 years of follow up [10].

Celiac disease associated small intestinal adenocarcinoma is thought to occur from adenoma-carcinoma sequence. It is most commonly characterized by proximal duodenal/jejunal localization. Hemorrhage or obstruction are typical presentations, with abdominal pain, weight loss, and anemia being diagnostic clues [4]. Endoscopy and tissue biopsy are usually sufficient to establish the diagnosis; however, if the tumor is not identified by endoscopy; CT, video capsule endoscopy, or double-balloon enteroscopy can be pursued [8]. Surgical resection of localized tumor with regional lymph node resection is the only curative treatment. In a retrospective study evaluating 217 patients with small bowel adenocarcinoma, the median overall survival time was 20 months. The five-year overall survival rate was 26% [11]. Median survival time in patients with jejunal/ileal cancers was found to be worse than those with duodenal cancers (24.5 versus 74.1 months respectively, p = 0.003) [5]. In a study comparing adjuvant chemotherapy to surgery alone, adjuvant chemotherapy showed a superior survival benefit in patients with AJCC stage III disease (42.4 versus 26.1 months p < 0.001), but failed to show benefit in stage I and II disease [12].

## Conclusions

Adenocarcinoma of the small intestine is a rare neoplasm but the risk is increased by environmental and genetic factors. CD increases the risk of developing small intestinal adenocarcinoma and there are no guidelines that support screening in this population due to rarity of this tumor; however, because of the overlap of symptoms, CD patients who are noncompliant with the gluten free diet and develop symptoms such as weight loss and abdominal pain might warrant testing with abdominal imaging or endoscopy.
